# Real‐world effectiveness and safety of bimekizumab in Japanese patients with psoriasis: A single‐center retrospective study

**DOI:** 10.1111/1346-8138.17186

**Published:** 2024-03-14

**Authors:** Teppei Hagino, Hidehisa Saeki, Eita Fujimoto, Naoko Kanda

**Affiliations:** ^1^ Department of Dermatology Nippon Medical School Chiba Hokusoh Hospital Inzai Japan; ^2^ Department of Dermatology Nippon Medical School Tokyo Japan; ^3^ Fujimoto Dermatology Clinic Funabashi Japan

**Keywords:** bimekizumab, biologic, interleukin‐17F, psoriasis, real‐world clinical practice

## Abstract

Bimekizumab, which suppresses both interleukin (IL)‐17A and IL‐17F, has recently been approved as a biologic for psoriasis. We aimed to evaluate the real‐world effectiveness and safety of bimekizumab for psoriasis and to identify predictive factors for its treatment responsiveness. We analyzed 36 Japanese patients with psoriasis (19 with psoriasis vulgaris and 17 with psoriatic arthritis) from May 2022 to September 2023. All patients received bimekizumab (320 mg every 4 weeks) until week 16. Seventeen patients (43.2%) had experienced bio‐switch. The median (interquartile range) baseline total psoriasis area and severity index (PASI) was 6 (3.2–20.0). Total PASI rapidly and significantly decreased at week 4 by a median 79.8% from baseline, and gradually decreased thereafter. The PASI on the trunk, and upper and lower limbs rapidly and significantly decreased at week 4 compared to baseline and plateaued thereafter. The neutrophil‐to‐lymphocyte ratio and neutrophil number significantly decreased at week 16 compared to baseline. At weeks 4, 8, 12, and 16, the achievement rate of absolute PASI ≤2 was 72.2%, 80.6%, 92.9%, and 96.4%, respectively; that of absolute PASI ≤1 was 41.7%, 61.3%, 85.7%, and 82.1%; that of PASI 75 was 55.5%, 52.9%, 69.7%, and 75.8%; that of PASI 90 was 36.1%, 50.0%, 57.6%, and 62.9%; and that of PASI 100 was 19.4%, 38.2%, 51.5%, or 57.6%, respectively. Linear multivariate regression analysis revealed that younger age was associated with a higher percentage reduction of total PASI at weeks 4 and 8. There were no serious or fatal adverse events during treatment. In conclusion, bimekizumab rapidly and remarkably reduced the total PASI together with high achievement rates of absolute PASI ≤1 and ≤2, and with favorable safety in real‐world clinical practice. Younger age may be a predictive factor for a good treatment response to bimekizumab.

## 1 INTRODUCTION

Psoriasis is a chronic, inflammatory skin disease with an enhanced interleukin (IL)‐23/IL‐17 immune axis.^1^ Various biologics or small molecule agents targeting this axis have been developed for the treatment of psoriasis. In total, 12 kinds of biologics (adalimumab, infliximab, certolizumab pegol, ixekizumab, secukinumab, brodalumab, bimekizumab, ustekinumab, guselkumab, risankizumab, tildrakizumab, and spesolimab) are currently approved for psoriasis treatment in Japan. In May 2022, bimekizumab, a humanized monoclonal antibody that binds both IL‐17A and IL‐17F, was approved in Japan for the treatment of patients with moderate‐to‐severe psoriasis aged ≥15 years. The clinical trials have demonstrated the efficacy and safety of bimekizumab,^2^, ^3^, ^4^ however, reports on its effectiveness and safety in real‐world clinical practice are limited.^5^

In particular, background factors of patients to predict treatment responses to bimekizumab, such as age, sex, body mass index (BMI), disease duration, baseline psoriasis area and severity index (PASI), presence or absence of bio‐switch, and arthritis, have not been precisely examined. In addition, laboratory parameters that reflect the therapeutic effects of bimekizumab are unknown. C‐reactive protein (CRP), neutrophil‐to‐lymphocyte ratio (NLR), monocyte‐to‐lymphocyte ratio (MLR), and platelet‐to‐lymphocyte ratio (PLR) have been identified as indicators of systemic inflammation and might reflect the severity of psoriasis.^6^, ^7^, ^8^, ^9^, ^10^ Thus, these laboratory parameters may act as biomarkers reflecting the therapeutic effects of bimekizumab.

In this study we aimed to assess the effectiveness and safety of bimekizumab in real‐world clinical practice and to detect predictive factors for treatment responses to bimekizumab in psoriasis patients.

## 2 METHODS

### 2.1 Study design and data collection

We analyzed the effectiveness and safety of bimekizumab in 36 adult Japanese psoriasis patients (19 with psoriasis vulgaris and 17 with psoriatic arthritis) treated with bimekizumab at the Department of Dermatology in Nippon Medical School Chiba Hokusoh Hospital from May 2022 to September 2023. All the patients received bimekizumab (320 mg every 4 weeks) until week 16. This study was conducted in accordance with the Declaration of Helsinki (2004) and was approved by the Ethics Committee of Nippon Medical School Chiba Hokusoh Hospital. Medical records of patients were analyzed retrospectively. The diagnosis of psoriasis was made clinically. Before treatment, we recorded the patients' sex, age, BMI, disease duration, presence or absence of bio‐switch and the number of occurrences, presence or absence of arthritis, scalp, nail or genital lesions, diabetes mellitus, and current smoking.

Total (whole body) PASI, PASI on the head and neck, trunk, and upper and lower limbs were analyzed at weeks 0, 4, 8, 12, and 16 of treatment. The values of NLR, MLR, PLR and CRP, numbers of neutrophils, lymphocytes, monocytes, and platelets were analyzed at weeks 0 and 16.

The achievement rates of PASI 75, 90, or 100 (i.e., the improvement of PASI from baseline ≥75%, ≥90%, or 100% respectively), of absolute PASI ≤2 or absolute PASI ≤1, and percentage reductions of PASI were calculated at weeks 4, 8, 12 and 16.

Safety was assessed by the occurrence of treatment‐emergent adverse events (TEAEs) during bimekizumab treatment, until 30 days after the last dose. A TEAE was defined as any adverse event (AE) that began or worsened after the initiation of treatment.

### 2.2 Statistical analysis

Results are expressed as the mean ± standard deviation for variables with a normal distribution, or as the median and interquartile range for variables with a non‐parametric distribution. Differences between weeks 0, 4, 8, 12, and 16 were analyzed using Friedman's test for variables with non‐parametric distribution. Bonferroni correction was used for post‐hoc analysis.

A comparison between two independent groups was performed using the Mann–Whitney *U* test for variables with non‐parametric distribution. A comparison between two groups was performed using Wilcoxon rank sum test for variables with non‐parametric distribution. The correlations of the variables with each other were analyzed using Spearman's correlation coefficients. Statistical significance was set at *p* < 0.05.

A linear multivariate regression analysis was performed to determine the predictive factors for a high percentage reduction of PASI at weeks 4, 8, 12, and 16 of bimekizumab treatment. The analysis included only the variables with a *p* value of <0.05 in univariate analyses and was adjusted for age, sex, and BMI. Variables with a variance inflation factor of >10 were excluded to avoid multicollinearity. All statistical analyses were performed using EZR (Saitama Medical Center, Jichi Medical School).

## 3 RESULTS

### 3.1 Patient demographics and baseline characteristics

Thirty‐six Japanese patients with psoriasis were enrolled in the study (Table 1). Baseline (week 0) values of the total PASI, PASI on four anatomical sites, NLR, MLR, PLR, CRP, neutrophil, lymphocyte, monocyte, and platelet numbers are shown in Table 1. The majority of patients were male (75.0%). The baseline total PASI was median (interquartile range) 6.0 (3.4–12.3). The low baseline PASI might be attributable to the high rate of pretreatment with other biologics, which might have reduced PASI scores before initiation of bimekizumab treatment. Seventeen patients (47.2%) had experienced bio‐switch; one patient was pretreated with adalimumab, two with infliximab, one with certolizumab pegol, one with ixekizumab, six with secukinumab, two with ustekinumab, five with guselkumab, one with risankizumab, and two patients with tildrakizumab.

**TABLE 1:** Baseline characteristics of psoriasis patients treated with bimekizumab (*n* = 36).

Male sex, *n* (%)	27 (75.0)
Age (years)^a^	59.5 (47.8–70.5)
Body mass index (kg/m^2^)^b^	24.8 ± 3.9
Disease duration (years)^a^	10.0 (3.0–21.3)
Positive biologic switch, *n* (%)	17 (47.2)
Number of bio‐switches, *n*	
1	14
2	2
5	1
Presence of arthritis, *n* (%)	17 (47.2)
Presence of scalp lesions, *n* (%)	33 (91.7)
Presence of nail lesions, *n* (%)	24 (66.7)
Presence of genital lesions, *n* (%)	22 (61.1)
Current smoking, *n* (%)	15 (41.7)
Diabetes mellitus, *n* (%)	4 (11.1)
Clinical indexes	
Total PASI^a^	6 (3.2–20.0)
PASI on head and neck^a^	0.2 (0–0.80
PASI on trunk^a^	1.8 (0.9–3.6)
PASI on upper limbs^a^	0.6 (0.6–2.4)
PASI on lower limbs^a^	3.2 (1.2–6.4)
Types of psoriasis, *n* (%)	
Psoriasis vulgaris	19 (52.8)
Psoriatic arthritis	17 (47.2)
Laboratory findings	
NLR^a^	2.14 (1.79–2.52)
MLR^a^	0.25 (0.20–0.35)
PLR^a^	133.3 (103.9–185.6)
CRP (mg/dL)^a^	0.065 (0.04–0.13)
Neutrophils (/μL)^a^	3299.4 (2539.8–4530.0)
Lymphocytes (/μL)^a^	1820.7 (1396.5–2432.0)
Monocytes (/μL)^a^	401.6 (335.4–504.1)
Platelets (10^3^/μL)^a^	237.0 (212.0–271.0)

Abbreviations: CRP, C‐reactive protein; MLR, monocyte‐to‐lymphocyte ratio; NLR, neutrophil‐to‐lymphocyte ratio; PASI, Psoriasis Area and Severity Index; PLR, platelet‐to‐lymphocyte ratio.

^a^ Data provided as median (interquartile range).

^b^ Data provided as mean ± standard deviation.

### 3.2 Transition of total PASI and PASI on individual anatomical sites during treatment with bimekizumab

**FIGURE 1:**
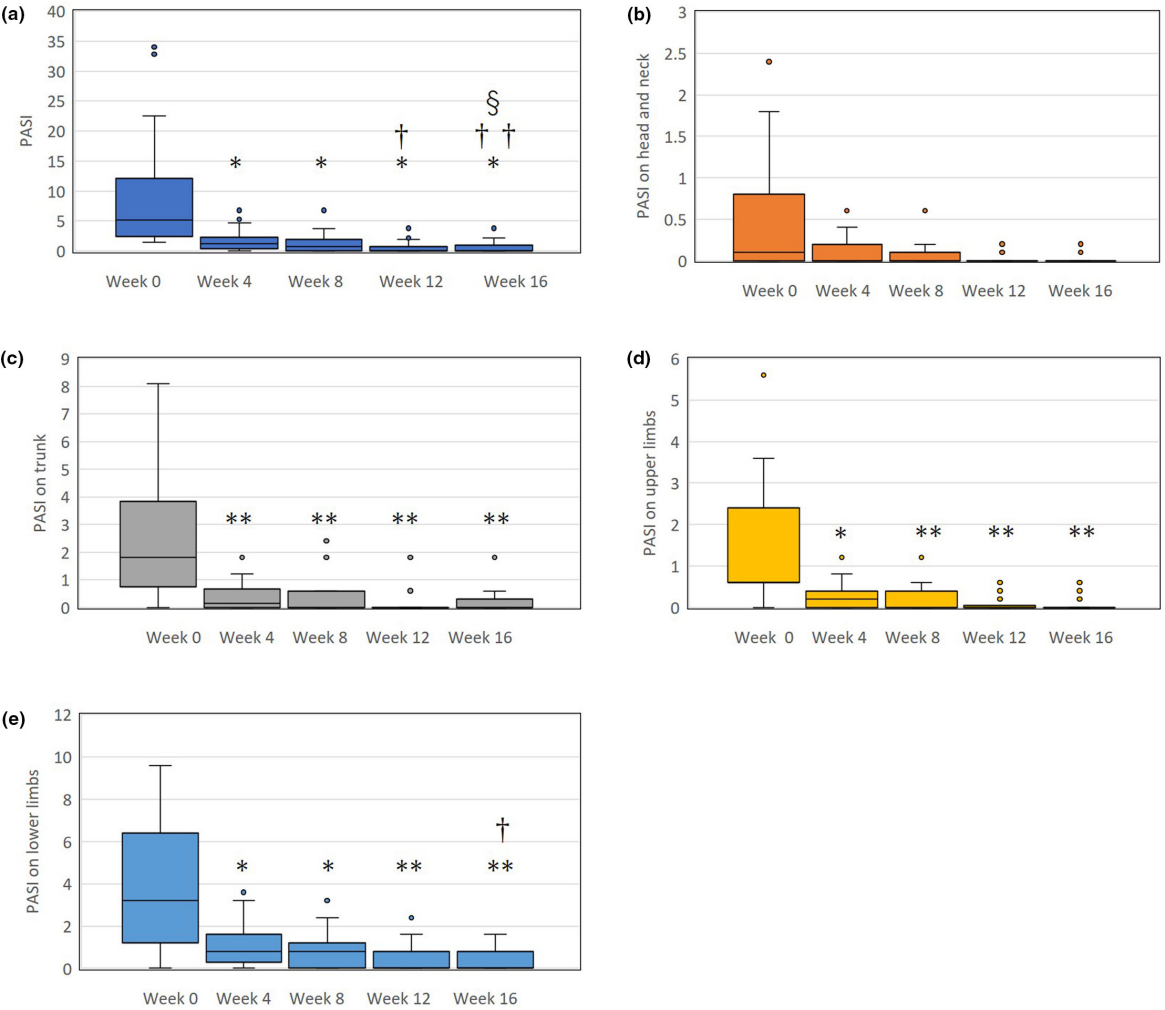
(a) The reduction of total psoriasis area and severity index (PASI) scores. (b) PASI scores on head and neck (c), trunk, (d) upper limbs, and (e) lower limbs during bimekizumab treatment in patients with psoriasis (*n* = 36). Data are provided as the median (interquartile range). *, *p* < 0.05, ** *p* < 0.01 versus values of week 0; †, *p* < 0.05, ††, *p* < 0.01 versus values of week 4; §, *p* < 0.05 versus values of week 8, assessed by Friedman's test with the Bonferonni post‐hoc test.

Total PASI rapidly and significantly reduced at week 4 by a median 79.8% of baseline, and gradually reduced thereafter (Figure 1a). The total PASI score at week 12 was significantly lower than that at week 4, and that at week 16 was significantly lower than those at weeks 4 and 8. The PASI score on the trunk and upper and/or lower limbs rapidly and significantly reduced at week 4 by a median 86.7%, 80.0%, or 66.7% of baseline respectively, and plateaued thereafter (Figure 1c–e). PASI on the head and neck appeared to decrease at week 4 by a median 73.2% of baseline, however, the difference from baseline was not significant (Figure 1b).

### 3.3 Changes of laboratory parameters after bimekizumab treatment, and correlations between percentage reductions of those parameters versus that of total PASI

The neutrophil‐to‐lymphocyte ratio and number of neutrophils at week 16 were significantly lower than those at week 0, while MLR, PLR, numbers of lymphocytes, monocytes, and platelets and CRP at week 16 were not significantly different from those at week 0 (Table 2). These results suggest that the reduction of NLR after bimekizumab treatment may be attributable to that of the number of neutrophils in the blood.

**TABLE 2:** The changes in laboratory parameters before and after bimekizumab treatment (*n* = 36).

	Median (interquartile range)		*p*
	Week 0	Week 16	Week 0 vs week 16
NLR	2.14 (1.79–2.52)	1.75 (1.37–2.06)	0.014*
MLR	0.25 (0.20–0.35)	0.21 (0.17–0.24)	0.256
PLR	133.3 (103.9–185.6)	133.3 (114.7–180.4)	0.486
CRP (mg/dL)	0.065 (0.04–0.13)	0.07 (0.06–0.23)	0.75
Neutrophils (/μL)	3299.4 (2539.8–4530.0)	2805.0 (2345.3–4017.6)	< 0.01**
Lymphocytes (/μL)	1820.7 (1396.5–2432.0)	1722.6 (1445.4–2063.1)	0.67
Monocytes (/μL)	401.6 (335.4–504.1)	360.7 (310.2–440.6)	0.338
Platelets (10^3^/μL)	237.0 (212.0–271.0)	241.5 (209.8–264.0)	0.776

Abbreviations: CRP, C‐reactive protein: MLR, monocyte‐to‐lymphocyte ratio; NLR, neutrophil‐to‐lymphocyte ratio; PLR, platelet‐to‐lymphocyte ratio.

* *p* < 0.05.

** *p* < 0.01, by Wilcoxon rank sum test.

We then analyzed if the percentage reductions of laboratory parameters at week 16 might be correlated with that of the total PASI score (Table 3). There were no significant correlations between the percentage reduction of total PASI with those of laboratory parameters except for a positive correlation with that of lymphocyte number. However, that correlation appeared non‐specific since the lymphocyte number at week 16 was not significantly reduced compared to baseline (Table 2).

**TABLE 3:** Correlations of percent reduction of total PASI with those of laboratory parameters at week 16 of bimekizumab treatment in patients with psoriasis (*n* = 36).

Laboratory parameters	Rho	*p*
NLR	−0.279	0.278
MLR	−0.411	0.101
PLR	−0.33	0.182
CRP (mg/dL)	0.0454	0.849
Neutrophils (/μL)	−0.194	0.457
Lymphocytes (/μL)	0.489	0.0463*
Monocytes (/μL)	−0.058	0.819
Platelets (10^3^/μL)	−0.0617	0.808

*Note*: Correlations between variables were examined using Spearman's correlation coefficient.

Abbreviations: CRP, C‐reactive protein; MLR, monocyte‐to‐lymphocyte ratio; NLR, neutrophil‐to‐lymphocyte ratio; PASI, psoriasis area and severity index; PLR, platelet‐to‐lymphocyte ratio.

* Statistically significant at *p* < 0.05.

### 3.4 Achievement rates of PASI 75, PASI 90, PASI 100, absolute PASI ≤2, and absolute PASI ≤1 during bimekizumab treatment

**FIGURE 2:**
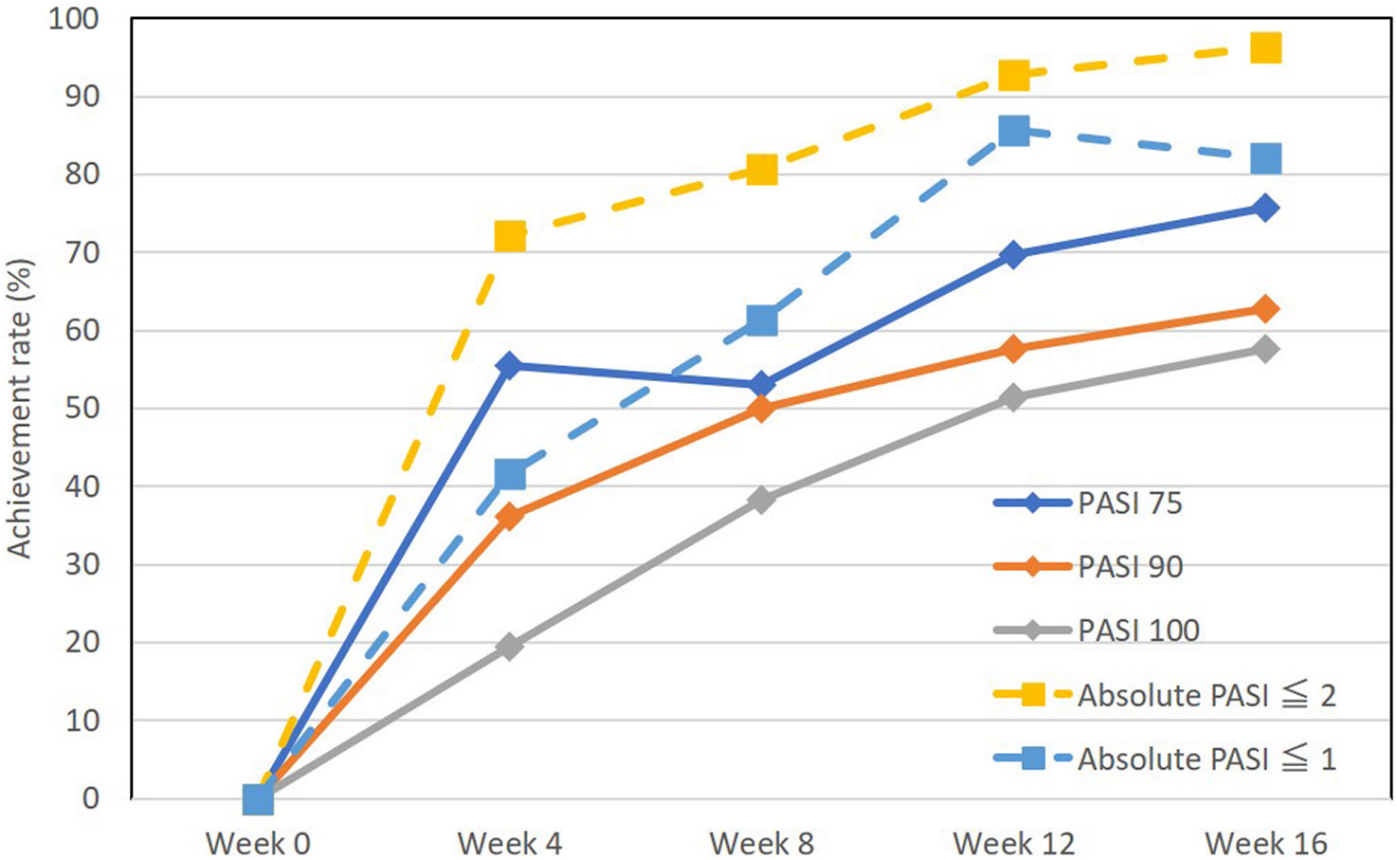
Achievement rates of psoriasis area and severity index (PASI) 75, PASI 90, PASI 100, absolute PASI ≤2, and absolute PASI ≤1 during bimekizumab treatment in patients with psoriasis (*n* = 36).

We evaluated the achievement rates of PASI 75, PASI 90, PASI 100, absolute PASI ≤2, and absolute PASI ≤1 during bimekizumab treatment in patients with psoriasis. At weeks 4, 8, 12, and 16, the achievement rate of PASI 75 was 55.5%, 52.9%, 69.7%, and 75.8% respectively; that of PASI 90 was 36.1%, 50.0%, 57.6%, or 62.9%; that of PASI 100 was 19.4%, 38.2%, 51.5%, and 57.6%, that of absolute PASI ≤2 was 72.2%, 80.6%, 92.9%, and 96.4%; and that of absolute PASI ≤1 was 41.7%, 61.3%, 85.7%, and 82.1%, respectively (Figure 2).

### 3.5 Background factors predicting treatment response to bimekizumab in patients with psoriasis

We tried to detect background factors that may predict a high treatment response to bimekizumab, as evaluated by the percentage reduction of the total PASI score. We first analyzed whether sex, presence or absence of bio‐switch, arthritis, scalp, nail, or genital lesions, diabetes, or current smoking affected the treatment response to bimekizumab. There were no differences in percentage reductions of total PASI between males and females, or between groups with versus groups without arthritis, scalp, nail, or genital lesions, diabetes, current smoking, or bio‐switch (Table 4). We next examined whether age, BMI, disease duration, or baseline values of total PASI or PASI on individual anatomical sites, or laboratory parameters affected the treatment responsiveness to bimekizumab. Patients' age was negatively correlated with the percentage reductions of total PASI at weeks 4 and 8 (Table 5), indicating that younger age may predict a higher treatment response to bimekizumab at weeks 4 and 8. Additionally, baseline PASI on upper limbs was positively correlated with the percentage reduction of PASI at week 4. The other variables were not significantly correlated with the percentage reductions of the total PASI score.

**TABLE 4:** The comparisons of percentage reductions of total PASI between two independent groups based on the background factors at weeks 4, 8, 12, or 16 of bimekizumab treatment in patients with psoriasis (*n* = 36).

Background factors	PASI reduction of at week 4			Percent reduction of PASI at week 8			Percent reduction of PASI at week 12			Percent reduction of PASI at week 16		
Sex (*n*)	Male (27)	Female (9)	*p*	Male (27)	Female (9)	*p*	Male (27)	Female (9)	*P*	Male (27)	Female (9)	*p*
	81.49 (58.0–93.2)	78.3 (68.3–86.7)	0.84	91.1 (64.0–100.0)	79.8 (66.9–100.0)	0.91	100.0 (80.6–100.0)	100.0 (86.7–100.0)	0.90	100.0 (93.7–100.0)	100.0 (86.7–100.0)	0.76
Biologic switch (*n*)	Absence (19)	Presence (17)	0.94	Absence (19)	Presence (17)	0.54	Absence (19)	Presence (17)	0.49	Absence (19)	Presence (17)	0.79
	80.0 (66.2–90.7)	73.1 (50.0–100.0)		91.2 (68.2–100.0)	87.0 (58.3–100.0)		100.0 (90.0–100.0)	100.0 (77.1–100.0)		100.0 (91.9–100.0)	100.0 (90.0–100.0)	
Arthritis (*n*)	Absence (19)	Presence (17)	0.92	Absence (19)	Presence (17)	0.81	Absence (19)	Presence (17)	0.51	Absence (19)	Presence (17)	0.69
	83.0 (58.0–95.6)	78.3 (66.7–91.1)		91.1 (64.0–100.0)	91.2 (67.1–100.0)		100.0 (88.9–100.0)	100.0 (86.7–100.0)		100.0 (90.2–100.0)	100.0 (91.1–100.0)	
Scalp lesions (*n*)	Absence (3)	Presence (33)	0.066	Absence (3)	Presence (33)	0.86	Absence (3)	Presence (33)	0.74	Absence (3)	Presence (33)	0.93
	100.0 (93.3–100.0)	78.3 (60.0–91.1)		100.0 (40.0–100.0)	91.1 (64.0–100.0)		100.0 (93.3–100.0)	100.0 (83.6–100.0)		100.0 (93.3–100.0)	100.0 (91.1–100.0)	
Nail lesions (*n*)	Absence (12)	Presence (24)	0.70	Absence (12)	Presence (24)	0.86	Absence (12)	Presence (24)	0.74	Absence (12)	Presence (24)	0.93
	83.3 (68.3–91.0)	75.7 (53.1–94.9)		100.0 (40.0–100.0)	91.1 (64.0–100.0)		10.0 (93.3–100.0)	100.0 (86.7–100.0)		100.0 (93.3–100.0)	100.0 (91.‐100.0)	
Genital lesions (*n*)	Absence (14)	Presence (22)	0.55	Absence (14)	Presence (22)	1.0	Absence (14)	Presence (22)	1.0	Absence (14)	Presence (22)	0.61
	81.6 (65.1–97.8)	79.8 (60.4–90.7)		91.1 (64.0–100.0)	91.2 (64.7–100.0)		100.0 (86.7–100.0)	100.0 (81.7–100.0)		100.0 (86.7–100.0)	100.0 (93.0–100.0)	
Current smoking (*n*)	Absence (21)	Presence (15)	0.54	Absence (231)	Presence (15)	0.35	Absence (21)	Presence (15)	0.24	Absence (21)	Presence (15)	0.74
	81.9 (64.0–91.1)	73.1 (50.5–93.2)		91.2 (68.3–100.0)	67.4 (57.5–100.0)		100.0 (91.1–100.0)	100.0 (56.7–100.0)		100.0 (91.1–100.0)	100.0 (87.1–100.0)	
Diabetes mellitus (*n*)	Absence (32)	Presence (4)	0.94	Absence (32)	Presence (4)	0.97	Absence (32)	Presence (4)	0.8	Absence (32)	Presence (4)	0.60
	80.7 (61.3–91.6)	76.5 (63.1–85.0)		91.1 (64.0–100.0)	80.6 (70.8–90.3)		100.0 (86.7–100.0)	90.3 (85.4–95.1)		100.0 (91.7–100.0)	90.3 (85.4–95.1)	

*Note*: Data are shown as the median (interquartile range), assessed by Mann–Whitney *U* test.

Abbreviation: PASI, Psoriasis Area and Severity Index.

**TABLE 5:** The correlations of baseline values of background factors with the percent reduction of total PASI at week 4, 8, 12, and 16 of bimekizumab treatment in patients with psoriasis (*n* = 36).

Background factors	Week 4		Week 8		Week 12		Week 16	
	Rho	*p*	Rho	*p*	Rho	*p*	Rho	*p*
Age (years)	−0.342	0.0413*	−0.367	0.0424*	−0.251	0.198	−0.351	0.0666
Body mass index (kg/m^2^)	−0.0534	0.757	0.12	0.521	0.0578	0.77	0.173	0.377
Disease duration (years)	−0.131	0.448	−0.0914	0.625	0.0622	0.753	0.175	0.374
Total PASI	0.0874	0.612	0.237	0.2	0.29	0.13	−0.0534	0.78
PASI on head and neck	−0.16	0.407	0.00652	0.976	0.00701	0.98	−0.32	0.16
PASI on trunk	−0.12	0.59	−0.0169	0.939	0.173	0.467	−0.259	0.27
PASI on upper limbs	0.376	0.044*	0.234	0.27	0.268	0.24	0.046	0.843
PASI on lower limbs	0.101	0.601	0.166	0.44	0.216	0.35	−0.141	0.54
NLR	−0.084	0.695	−0.196	0.395	−0.106	0.658	−0.405	0.076
MLR	−0.0197	0.927	−0.16	0.487	−0.0825	0.729	−0.434	0.056
PLR	−0.073	0.735	−0.104	0.655	0.111	0.643	−0.16	0.501
CRP (mg/dL)	0.0101	0.963	−0.0539	0.817	−0.194	0.413	−0.133	0.576
Neutrophils (/μL)	−0.0641	0.807	0.0115	0.965	−0.0705	0.788	−0.215	0.408
Lymphocytes (/μL)	0.09	0.731	0.0547	0.835	−0.0997	0.703	0.279	0.278
Monocytes (/μL)	0.133	0.6	0.0213	0.933	−0.0852	0.737	−0.131	0.605
Platelets (10^3^/μL)	−0.00437	0.984	−0.131	0.572	−0.0165	0.945	−0.161	0.498

*Note*: Correlations between variables were examined using Spearman's correlation coefficient.

Abbreviations: CRP, C‐reactive protein; MLR, monocyte‐to‐lymphocyte ratio; NLR, neutrophil‐to‐lymphocyte ratio; PASI, psoriasis area and severity index; PLR, platelet‐to‐lymphocyte ratio.

* Statistically significant at *p* < 0.05.

Next, linear multivariate regression analysis was performed to determine the predictive factors for the treatment response to bimekizumab, as evaluated by the percentage reduction of the total PASI score. A high percentage reduction of total PASI score was associated with younger age at weeks 4 and 8 of treatment with bimekizumab (Table 6). The results indicate that younger age may be a predictive factor for a high treatment response to bimekizumab at weeks 4 and 8 in patients with psoriasis.

**TABLE 6:** The predictive factors for the percentage reduction of total PASI scores at weeks 4 or 8 of bimekizumab treatment assessed by linear multivariate regression analysis in patients with psoriasis (*n* = 36).

	Week 4				Week 8			
	β coefficient	Standard error	*t*	*p*	β coefficient	Standard error	*t*	*p*
Intercept	121.5	40.8	3.0	0.0065	119.3	49.4	2.491	0.022
Age (years)	−0.75	0.24	−3.1	< 0.01**	−0.76	0.29	−2.6	0.015*
Sex	14.4	10.8	1.3	0.20	−2.97	13.23	−0.22	0.82
Body mass index (kg/m^2^)	−1.4	1.1	−1.28	0.21	0.13	1.58	0.082	0.94
PASI on upper limbs	5.8	2.9	2.0	0.06	NA			

Abbreviation: PASI, Psoriasis Area Severity Index.

* Statistically significant at *p* < 0.05.

** At *p* < 0.01.

Our extended analyses, detailed in Table S1, compared baseline characteristics between responders and non‐responders at week 16, where responders were defined by the achievement rate of PASI 100. Although a significant difference in MLR was observed, further logistic regression analysis (Table S2) did not identify MLR or any other background factor as significantly correlated with the achievement rate of PASI 100 at week 16. This indicates that the predictors of response to bimekizumab in our study might be influenced by factors beyond those captured in the study. These results emphasize the complexity of predicting treatment response and highlight the need for further, large‐scale studies to identify more nuanced predictive factors, particularly in specific subgroups such as the elderly.

### 3.6 Safety profile

The safety profile for bimekizumab in this real‐world study (Table 7) was similar to that in the previous clinical trial.^11^ No new AEs were observed with bimekiuzmab. TEAEs occurred in 12 patients (33.3%). There were no serious AEs or AEs that resulted in death. Major AEs were pruritus in three patients (8.3%) and oral candidiasis in three patients (8.3%), which was reported as a frequent AE during bimekizumab treatment.^2^, ^3^, ^4^ Tinea pedis occurred in two patients (5.6%), exfoliation of the palmoplantar skin in two patients (5.6%), fatigue in one patient (2.8%) which resolved without any specific treatment, and exacerbation of psoriatic arthritis in one patient (2.8%). These events were mild in severity and improved with appropriate medicine. One AE (2.8%) leading to discontinuation of bimekizumab was exacerbation of associated atopic dermatitis. This case was improved by treatment with Janus kinase 1/2 inhibitor, baricitinib (4 mg/day), and topical corticosteroids.

**TABLE 7:** Treatment‐emergent adverse events (TEAEs) until week 16 of treatment with bimekizumab in patients with psoriasis (*n* = 36).

TEAE	*n* (%)
All TEAEs	12 (33.3)
Serious AE	0
AE leading to discontinuation of bimekizumab	1 (2.8)
AE leading to death	0
AEs of special interest	
*Tinea pedis*	2 (5.6)
Exfoliation of the palmoplantar skin	2 (5.6)
Fatigue	1 (2.8)
Exacerbation of psoriatic arthritis	1 (2.8)
Pruritus	3 (8.3)
Oral candidiasis	3 (8.3)
Exacerbation of atopic dermatitis	1 (2.8)

Abbreviation: AE, adverse event.

## 4 DISCUSSION

In this study, whole body rash of psoriasis, as evaluated by total PASI, rapidly and remarkably improved by 79.8% of baseline at week 4 of bimekizumab treatment and almost completely disappeared at weeks 12 or 16. The high therapeutic effectiveness at an early time‐point might be attributable to the dual inhibition of IL‐17A and IL‐17F by bimekizumab. By selectively binding to IL‐17F as well as IL‐17A and IL‐17A/F, bimekizumab suppresses the activation of receptor complex IL‐17RA/RC by these cytokines and the subsequent signaling cascade. The dual blockade of IL‐17A and IL‐17F may be more effective than IL‐17A blockade alone. IL‐17F induced qualitatively similar inflammatory responses to IL‐17A in dermal fibroblasts and synoviocytes, such as secretion of IL‐6 and IL‐8, though they were quantitatively less potent.^12^ Compared to selective inhibition of IL‐17A or IL‐17F alone, neutralization of IL‐17A and IL‐17F with bimekizumab more effectively suppressed in vitro production of cytokines/chemokines, such as IL‐6 or the chemokine (C‐X‐C motif) ligand 1 (CXCL1)/2/8 in fibroblasts/synoviocytes^12^ and keratinocytes,^13^ and neutrophil chemotaxis induced by T helper 17 (Th17) cell supernatants.^12^ Bimekizumab treatment completely reversed the levels of cytokines/chemokines, anti‐microbial peptides, or proliferation‐related molecules, such as CXCL8, CCL20, IL‐17A, IL‐17F, IL‐17C, keratin 16, IL‐36γ, DEFB4, or S100A7, in psoriasis lesional skin to the levels of non‐lesional skin.^13^ Those in vitro and in vivo effects of bimekizumab may be reflected by the almost complete disappearance of eruptions at weeks 12 or 16 of bimekizumab treatment in this study.

In parallel with whole body rash, rash on individual anatomical sites, as evaluated by PASI, rapidly and remarkably improved at week 4 of bimekizumab treatment. These results indicate that bimekizumab might equally improve the rash on individual anatomical sites. The lack of a significant difference in PASI scores on the head and neck between, before, and after treatment (Figure 1b) may be due to the very low baseline scores of PASI on these regions.

In this real‐world clinical study, the achievement rates of PASI 75, 90, and 100 appeared lower than those reported in past clinical trials.^2^, ^3^, ^4^ The achievement rates at week 16 of our study versus clinical trials were 75.8% versus 95.4% for PASI 75, 62.9% versus 90.8% for PASI 90, 57.6% versus 68.2% for PASI 100, respectively. In contrast, the achievement rates of absolute PASI ≤2 or ≤1 in this study appeared higher than those reported in past clinical trials: 96.4% versus 86.5% for absolute PASI ≤2, 82.1% versus 80.5% for absolute PASI ≤1 at week 16 in this study versus clinical trials, respectively. Such discrepancy between achievement rates of PASI 75, 90, 100 versus absolute PASI ≤2 or ≤1 may possibly be due to the lower baseline scores of PASI in this real‐world study since percentage change of PASI is largely dependent on baseline absolute PASI scores.^14^ A high proportion (47.2%) of participants in our study had been pretreated with other biologics and without sufficient wash‐out time before bimekizumab treatment, which might contribute to the low baseline absolute PASI scores. Bimekizumab‐treated patients were significantly more likely to achieve PASI 90 or PASI 100 within 10–16 weeks of the first injection than patients treated with all other biologics in a network meta‐analysis.^15^ Clinicians might hesitate to select bimekizumab as a first biologic and tend to hold it as a second or third biologic, considering that patients who have experienced extremely high therapeutic effects of bimekizumab might be dissatisfied with the other biologics. This might be the possible reason for the low rate of bio‐naive patients, namely the high rate of bio‐switched patients in bimekizumab‐treated cohort in this real‐world study.

After 16 weeks of bimekizumab treatment, NLR and the blood neutrophil number significantly reduced, indicating that bimekizumab might suppress neutrophilia. IL‐17A and IL‐17F promote neutrophilia. Both cytokines induce granulopoiesis in bone marrow via induction of granulocyte‐colony stimulating factor secretion in bone marrow stromal cells.^16^, ^17^ IL‐17A also increases viability of blood neutrophils by suppressing their apoptosis.^18^ The reduction of the neutrophil number and NLR after bimekizumab treatment might be mediated by suppressing the above effects of IL‐17A and IL‐17F. These effects of bimekizumab might warn of the occurrence of neutropenia during the treatment. In this study, serious neutropenia (neutrophil number <1000/mm^3^) did not occur. However, clinicians should monitor the occurrence of neutropenia with regular blood tests during bimekizumab treatment. The percentage reductions of NLR and neutrophil number were not significantly correlated with that of total PASI at week 16. The correlation should be further investigated by a large‐scale study with more frequent examination during bimekizumab treatment to see if NLR and/or neutrophil number can function as a biomarker reflecting the therapeutic effects of bimekizumab.

Multivariate regression analysis showed that high percentage reductions of PASI by bimekizumab treatment at weeks 4 and 8 were associated with younger age, indicating that younger age might predict a better treatment response to bimekizumab. It is reported that healthy elderly people (aged ≥65 years) have increased Th17 frequency and retinoic acid receptor‐related orphan receptor γt expression in peripheral blood mononuclear cells and increased serum levels of IL‐17A and IL‐6 compared to healthy middle‐aged (aged 45–64 years) and young people (aged ≤44 years), indicating that IL‐17‐related immune response is enhanced with aging.^19^ It is thus hypothesized that younger psoriasis patients may have lower IL‐17A, IL‐17A/F, or IL‐17F‐mediated immune responses, which may be more susceptible to bimekizumab treatment compared to elderly patients.

Other than age, patients' sex, BMI, presence of arthritis, scalp, nail, or genital lesions, diabetes, current smoking, or prior bio‐switch did not appear to influence the treatment responses to bimekizumab as evaluated by the percentage reduction of total PASI. The results indicate that bimekizumab might be universally effective for psoriasis patients, independently of various background factors, except for age.

The safety profile of bimekizumab in this real‐world study was akin to that in a previous clinical trial.^11^ In this study, there were three cases of oral candidiasis (8.3%), which appeared to be lower than the frequency in the clinical trial (15.6% or 19.3%)^2^, ^4^ possibly due to the small sample size and/or short duration of observation in this study. IL‐17A is necessary for defense against *Candid albicans* by promoting recruitment of neutrophils via IL‐8 production in epithelial cells. IL‐17F is produced by oral γδT cells during oropharyngeal candidiasis, and might contribute to defense against *Candid albicans*, though less potently than IL‐17A.^20^ Mice with homozygous mutation of IL‐17F (*Il17f* ^*S65L/S65L*^) showed increased oral burdens of *Candid albicans*, and showed modestly impaired CXCL1 expression and neutrophil recruitment to the infected tongue.^20^ IL‐17A–neutralizing antibody exhibits elevated susceptibility to oropharyngeal candidiasis^21^ while IL‐17F–neutralizing antibody does not alter the susceptibility; however, dual blockade of IL‐17A and IL‐17F increases susceptibility to oropharyngeal candidiasis over blockade of IL‐17A alone.^21^ Similar mechanisms might be anticipated for the higher frequency of oral candidiasis during treatment with bimekizumab compared to the other antibodies specific to IL‐17A alone.

This study has several limitations. First, the baseline scores of total PASI were low. This might influence the achievement rates of PASI 75, 90, or 100. Second, the sample size was small. Third, medications for comorbidities or previous treatments for psoriasis were not included in the analysis. Fourth, we used the conventional method for the measurement of CRP. High‐sensitivity CRP quantification might be preferable.

In conclusion, this study demonstrated that bimekizumab rapidly and remarkably reduced total PASI scores at week 4, with high achievement rates of absolute PASI ≤2 and absolute PASI ≤1, and a favorable safety profile. Younger age might be a predictive factor for high treatment responses to bimekizumab.

## CONFLICT OF INTEREST STATEMENT

Teppei Hagino, Hidehisa Saeki, and Naoko Kanda received lecture fees from UCB Japan. The other authors have conflicts of interest to declare.

## Supporting information


Table S1.

Table S2.
