# Supplementary material for: Real‐world effectiveness and safety of bimekizumab in Japanese patients with psoriasis: A single‐center retrospective study
Source: J Dermatol. 2024 Mar 14;51(5):649–58. doi: 10.1111/1346-8138.17186 (PMC11484122; doi:10.1111/1346-8138.17186)
Supplement: Supplementary file 1 — Table S1. [file JDE-51--s002.docx]

| Supplemental table 1. The relations of responders and non-responders with background factors in patients with psoriasis (*n* = 33). | | | |
| --- | --- | --- | --- |
|  | Responders (19) | Non-responders (14) | *p* |
| Male sex^a^ | 15 (78.9) | 9 (64.3) | 0.442 |
| Age (years) ^b^ | 60 [40.5-71] | 61 [58.3-71.3] | 0.382 |
| Body mass index (kg/m²) ^c^ | 25.3 ± 4.2 | 24.9 ±3.5 | 0.801 |
| Disease duration (years)^b^ | 10 [3.5-23.0] | 8 [1.5-19.5] | 0.421 |
| Positive biologic switcha | 11 (57.9) | 6 (42.9) | 0.491 |
| Presence of arthritis ^a^ | 9 (47.4) | 7 (50.0) | 1 |
| Presence of scalp lesions ^a^ | 17 (89.5) | 13 (92.9) | 1 |
| Presence of nail lesions ^a^ | 14 (73.7) | 7 (50.0) | 0.273 |
| Presence of genital lesions^a^ | 11 (57.9) | 9 (64.3) | 1 |
| Current smoking ^a^ | 8 (42.1) | 6 (42.9) | 1 |
| Diabetes mellitus | 1 (5.3) | 3 (21.4) | 0.288 |
| Total PASI ^b^ | 4.7 [2.4-11.8] | 5.6 [4.5-11.2] | 0.5 |
| PASI on head and neck ^b^ | 0 [0-0.23] | 0.45 [0.075-0.9] | 0.0614 |
| PASI on trunk ^b^ | 0.9 [0.9-1.75] | 3.0 [1.05-3.6] | 0.142 |
| PASI on upper limbs ^b^ | 0.6 [0.6-1.9] | 1.2 [0.6-2.1] | 0.599 |
| PASI on lower limbs ^b^ | 3.2 [0.1-6.9] | 3.2 [2.2-6.1] | 0.587 |
| NLR ^b^ | 1.96 [1.79-2.31] | 2.31 [2.06-3.71] | 0.206 |
| MLR ^b^ | 0.21 [0.20-0.25] | 0.35 [0.28-0.37] | 0.0153* |
| PLR ^b^ | 129.4 [95.2-140.1] | 210.0 [127.9-259.1] | 0.0816 |
| CRP (mg/dL) ^b^ | 0.06 [0.05-0.11] | 0.095 [0.04-0.56] | 0.514 |
| Neutrophils (/μL) ^b^ | 3264.3 [2719.4-4831.0] | 4413.2 [2116.4-4530.0] | 0.712 |
| Lymphocytes (/μL) ^b^ | 1871.4 [1592.6-2432.0] | 987.9 [987.9-2520.0] | 0.154 |
| Monocytes (/μL) ^b^ | 401.6 [329.4-465.6] | 405.7 [362.6-603.6] | 0.673 |
| Platelets (10³/μL) ^b^ | 228.5 [212.0-255.8] | 271.0 [252.0-286.3] | 0.108 |
| ^a^ Data provided as *n* (%), assessed by Fisher’s exact test.  ^b^ Data provided as the median [interquartile range], assessed by Mann–Whitney U test.  ^c^ Data provided as the mean ± standard deviation, assessed by student’s t test.  * Statistically significant at *p* < 0.05.  PASI, psoriasis area and severity index; NLR, neutrophil-to-lymphocyte ratio; MLR, monocyte-to-lymphocyte ratio; PLR, platelet-to-lymphocyte ratio; CRP, C-reactive protein. | | | |
