# Supplementary material for: Real‐world effectiveness and safety of bimekizumab in Japanese patients with psoriasis: A single‐center retrospective study
Source: J Dermatol. 2024 Mar 14;51(5):649–58. doi: 10.1111/1346-8138.17186 (PMC11484122; doi:10.1111/1346-8138.17186)
Supplement: Supplementary file 2 — Table S2. [file JDE-51--s001.docx]

| Supplemental table 2. Multiple logistic regression analysis for the association of each variable with responders to treatment with bimekizumab for psoriasis (*n* = 33) | | | |
| --- | --- | --- | --- |
|  | Odds Ratio | 95% Confidential Interval | *p* |
| (Intercept) | 11200000 | 0.000275-4.6x10^16^ | 0.193 |
| Age (years) | 0.91 | 0.781-1.06 | 0.227 |
| Sex (M=1, F=2) | 0.27 | 0.0136-5.35 | 0.39 |
| Body mass index (kg/m^2^) | 0.836 | 0.505-1.38 | 0.485 |
| MLR | 0.000000821 | 0.0000000000000576-11.7 | 0.0955 |
| PASI, psoriasis area and severity index; MLR, monocyte-to-lymphocyte ratio. | | | |
